# Word-selective EEG/MEG responses in the English language obtained with fast periodic visual stimulation (FPVS)

**DOI:** 10.1162/imag_a_00414

**Published:** 2025-01-03

**Authors:** Olaf Hauk, Marion Marchive, Angelique Volfart, Christine Schiltz, Bruno Rossion, Matthew A. Lambon Ralph, Aliette Lochy

**Affiliations:** MRC Cognition and Brain Sciences Unit, University of Cambridge, United Kingdom; Institute of Cognitive Science and Assessment, University of Luxembourg, Esch-sur-Alzette, Luxembourg; Université de Lorraine, CNRS, IMoPA UMR 7365, Nancy, France; School of Psychology and Counselling, Faculty of Health, Queensland University of Technology, Brisbane, Australia; Université de Lorraine, CHRU-Nancy, Service de Neurologie, Nancy, France; Research Institute for Psychological Science, University of Louvain, Louvain-la-Neuve, Belgium

**Keywords:** visual word recognition, frequency tagging, magnetoencephalography, electroencephalography, source estimation

## Abstract

Fast periodic visual stimulation (FPVS) allows the objective measurement of brain responses of human word discrimination (i.e., reproducible word-category-selective responses) with a high signal-to-noise ratio. This approach has been successfully employed over the last decade in a number of scalp electroencephalography (EEG) studies. Three important advances for research on word-selective brain responses were achieved in the present study: (1) we extend previous evidence of robust word-category-selective responses to the English language, (2) report results for combined EEG and MEG signals, and (3) source estimation results. English words were presented periodically (2 Hz) among different types of letter strings (10 Hz; consonant strings, non-words, pseudo-words) while recording simultaneous EEG and MEG in 25 participants who performed a simple non-linguistic colour detection task. Data were analysed in sensor and in source space. With only 4 minutes of stimulation, we observed a robust word discrimination response in each condition, even when words were embedded in sequences of word-like pseudo-words. This response was larger in non-words and largest in consonant strings. We observed left-lateralised responses in all conditions in the majority of our participants. Cluster-based permutation tests revealed that these responses were left-lateralised in sensor as well as in source space, with peaks in left posterior regions. Our results demonstrate that the FPVS approach can elicit robust English word discrimination responses in EEG and MEG within only a few minutes of recording time. Together with source estimation, this can provide novel insights into the neural basis of visual word recognition in healthy and clinical populations.

## 1 Introduction

Recognising written words at a single glance is a crucial marker of reading expertise. Most adults in literate societies read fluently and effortlessly, often unaware of how complex this ability is at the cognitive level. In natural reading, a normal reader can discriminate and identify words at a rate of several words per second, offering the opportunity to present a large number of stimuli in a short amount of time. Here, we used a fast periodic visual stimulation (FPVS) paradigm to study EEG/MEG brain responses that discriminate between words and different types of non-word letter strings.

Over the past decade, FPVS with EEG or MEG has been used to investigate visual word recognition processes in adults (e.g.,Lochy et al., 2014,^2015^,^2018^;Pescuma et al., 2022) or in developmental studies (de Ghelcke et al., 2020,^2021^;Lutz et al., 2024;Wang et al., 2024,^2023^). The advantages of the FPVS paradigm over conventional (i.e., slow, non-periodic) event-related subtraction paradigms have been described previously, and include the objectivity of the dependent variable as a signal amplitude at an a priori-specified frequency, and a particularly high signal-to-noise ratio (SNR) within only a few minutes of recording time (Rossion et al., 2015).

Coupled with electroencephalography (EEG), FPVS measures word-selective neural responses over or in the left occipito-temporal cortex (Lochy et al., 2015,^2018^). In an oddball version of the paradigm, words are presented periodically (e.g., every five items) as deviants (oddballs) in streams of base stimuli (e.g., pseudo-words) displayed at a certain frequency (e.g., 10 Hz). If words are discriminated by the visual word recognition system from the base stimuli, then a word-selective response occurs exactly at the predefined oddball frequency (e.g., in base stimuli displayed at 10 Hz, words occur at 10/5 Hz, and responses are detectable at 2 Hz (Lochy et al., 2015,^2024^)). Importantly, the word-selective response represents a measure of differential processing between base and oddball stimuli, and, therefore, in the case of words among pseudo-words, it reflects the neural response to words over and above the responses that words and pseudo-words have in common. Thus similar to the logic used in some behavioural studies of lexical decision (e.g.,Evans et al., 2012), the oddball response is constrained by the contrast experimentally defined between the two stimulus categories, and provides a measure of coarse discrimination when words are inserted among pseudo-letters, while fine-grained pre-lexical or lexical-semantic discrimination is measured when words appear among non-words or pseudo-words (Lochy et al., 2015). Importantly, a recent study has shown that word-selective FPVS responses are truly linguistic in nature and, at least with a relatively high number of variable items used, are not explained by statistical learning due to different repetition rates of oddball and base stimuli (Lochy et al., 2024).

In spite of these clear observations and the significant advantages of the approach, its extension to English remains uncommon and sometimes elusive. In addition to the dearth of FPVS word recognition studies in general, one recent study with 10 participants encountered difficulties replicating the significant results of the original French paradigm in English (Barnes et al., 2021). This could be due to linguistic differences between French and English, or to different methodological choices (seeLochy et al. (2024), and discussion of the present study). Therefore, the application of FPVS to English word recognition needs further development before it can serve as an individually sensitive measure of written word processing in basic and clinical research. This was a major goal of the present study.

In addition, our study addresses two important methodological gaps in the FPVS literature: to date, word-selective responses have only been reported in EEG or MEG, but not for combined recordings, and without source estimation. Possible future applications of the word-selective FPVS paradigm include the fast determination of lateralized brain responses to linguistic stimuli. However, it is well known that sensor–space signals are difficult, if not impossible, to relate to putative brain regions without very strong prior information, in particular for EEG (e.g.,Ahlfors et al., 2010). The highest spatial resolution can be obtained using a combination of EEG and MEG recordings, as they are sensitive to different spatial aspects of the neural current distributions (Ahlfors et al., 2010;Goldenholz et al., 2009;Hauk et al. 2022;Henson et al., 2009;Molins et al., 2008). Thus, we evaluated the relative sensitivities of EEG and MEG sensor types as well as source estimates to word-selective FPVS responses using English stimulus material, particularly with respect to hemispheric laterality.

## 2 Methods

### 2.1 Participants

Twenty-nine participants were initially recruited from the MRC CBU’s volunteer panel database. Data from 4 of them had to be excluded because of technical problems or high noise levels, leaving 25 datasets for the final analysis. Of those, 14 identified as females. Mean age was 27 years (SD 6 years, range 19–40 years). All participants reported to be right-handed native English speakers, to have normal or corrected-to-normal vision, and to have no history of neurological or developmental disorders. They were monetarily reimbursed for their participation to the study. This study was approved by the Cambridge Psychology Research Ethics Committee.

In order to determine our sample size, we initially performed a power analysis based onLochy et al. (2015), which revealed a Cohen’s d for the word/pseudo-word contrast of 0.89. Using pwr.t.test in the R software package for a standard power of 80%, this yielded n = 12. Two previous related studies used n = 10 (Barnes et al., 2021;Lochy et al., 2015). Our sample size of n = 25, therefore, goes well beyond previous studies, and was ultimately determined by practical considerations about the available resources.

### 2.2 Stimuli

Our stimuli and experimental settings were close to those described inLochy et al. (2015). We used 30 high-frequency English nouns, each consisting of 5 letters (average frequency: 81.79 per million ± 139.57). These words had an average of 3.4 (± 2.69) orthographic neighbours and an average bigram frequency of 10,335 (± 3,259), calculated using Wordgen (Duyck et al., 2004). As a control condition, we aimed to have a coarse stimulus contrast that still relies on the visual recognition of the word. Thus, we constructed 120 5-letter consonant strings by randomly mixing consonants (independent from non- and pseudo-words) without referring to any linguistic or orthographic rules. Within each consonant string, each letter could only be repeated twice. For each of the 30 words, we also generated 4 corresponding 5-letter pseudo-words that matched the original in consonant/vowel structure, bigram frequency (9,279 ± 2,563), and number of orthographic neighbours (2.48 ± 1.85).

These pseudo-words were pronounceable and respected the phonological rules of the English language, but did not correspond to real words. They did not differ to the words with respect to bigram frequency (U = 1424, p = 0.078 with Mann–Whitney correction) and orthographic neighbours (U = 1454, p = 0.100 with Mann–Whitney correction). The set of 120 non-words was created by shuffling the letters of the pseudo-words, hence containing 5 letters that made them unpronounceable and did not follow the orthographic rules in English. Compared with words, and as expected, these non-words were significantly different in terms of bigram frequency (mean 4159 ± 2412) (t(148) = -11.637, p < 0.001) and orthographic neighbours (mean 0 ± 0).

All stimuli were presented on a grey background in a black Verdana font and varied randomly in size spanning 80% to 120% of their average size. They were shown on a screen at a distance of approximately 1.3 m in front of the participant. The stimuli subtended a maximum 4° of visual angle.

### 2.3 Procedure

A schematic illustration of the FPVS paradigm is presented inFigure 1. Stimuli were presented to participants using Java 8. All stimuli were presented on a uniform grey background using sinusoidal contrast manipulation, from 0 to 100% to 0% for each stimulus (Rossion et al., 2015). Monitor refresh rate was 60 Hz. Participants completed 4 runs of letter-string stimulation for each contrast (i.e., a contrast with either consonant strings, non-words or pseudo-words) leading to a total of 12 runs. Each run consisted of letter strings presented at a base frequency of 10 Hz (100 ms per stimulus), and every fifth image was a word (“oddball” frequency 2 Hz). A neural response that does not differ systematically between words and other types of letter strings will project on the 10 Hz component of the EEG/MEG spectrum and its harmonics (20 Hz, 30 Hz, etc.), whereas a differential response for words and non-word stimuli (i.e., a word-selective response) will be reflected at 2 Hz and harmonics (4 Hz, 6 Hz, etc.). Each run lasted for 64 seconds, including 2 seconds of fade-in and fade-out at the beginning and at the end of the sequence, respectively.

**Fig. 1.**
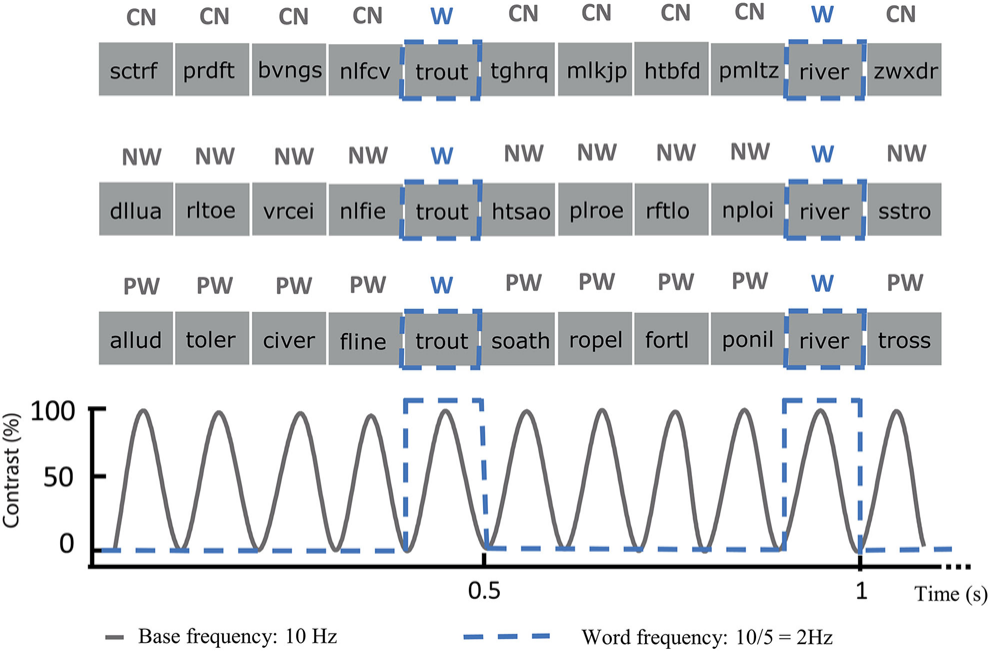
Illustration of the FPVS words paradigm. Consonant strings (CN), non-words (NW), or pseudo-words (PW) stimuli were presented at base frequency of 10 Hz using a sinusoidal contrast modulation. Words were inserted every 5 stimuli, corresponding to a word-selective frequency of 2 Hz (= 10 Hz/5).

Each sequence was presented randomly, and the stimuli as well (only the appearance of words every five images was controlled, but the word itself was selected randomly from our stimulus pool). The words were identical across the four conditions, and the base stimuli changed depending on the condition. However, in each case, the order of stimuli generated within each category (oddball/base) was random.

As in previous studies (e.g.,Rossion et al., 2015), participants were instructed to perform a colour change detection task during the stimulus presentation period. The stimuli were presented together with two lateral vertical bars that changed colour simultaneously or in isolation. Participants were asked to press a button with their right index finger when both bars changed from blue to red at the same time. This occurred randomly 8 times per run and lasted for 200 ms. The minimum difference in time between each colour change was 2 seconds. This task was chosen because it is orthogonal to the experimental manipulation (i.e., different types of letter strings), and has recently been shown to produce larger word-selective responses than a previously used central cross colour-change detection task (Lochy et al., 2024). Its purpose is to ensure that participants pay attention to the stimuli. This was confirmed, as performance was high with an average correct target detection rate of 98% (standard deviation 3%, min|max 89%|100%) and an average response time of 459 ms (SD 66 ms, min|max 347 ms|597 ms).

### 2.4 Data acquisition

EEG/MEG data were acquired on an Elekta Neuromag Triux neo system (Elekta AB, Stockholm, Sweden), containing 306 MEG sensors (102 magnetometers and 204 gradiometers), and 64 EEG electrodes mounted on an Easycap cap (EasyCap GmbH, Herrsching, Germany). The EEG recording reference electrode was attached to the nose, and the ground electrode to the left cheek. The electrooculogram (EOG) was recorded from electrodes above and below the left eye (vertical EOG) and at the outer canthi (horizontal EOG). The sampling rate during data acquisition was 1000 Hz and an on-line band pass filter 0.03 to 330 Hz was applied. Prior to the EEG/MEG recording, the positions of 5 Head Position Indicator (HPI) coils were attached to the EEG cap (for head localisation inside the scanner and continuous movement tracking), 3 anatomical landmark points (2 preauricular points and nasion) as well as the EEG electrodes and about 50–100 additional points that cover most of the scalp were digitised using a 3Space Isotrak II System (Polhemus, Colchester, Vermont, USA) for later co-registration with MRI data. Our data can be made available via the MRC Cognition and Brain Sciences Unit’s data repository on request.

High-resolution structural T1-weighted MRI images were acquired in a 3T Siemens Tim Trio scanner at the MRC Cognition and Brain Sciences Unit (UK) with a 3-D magnetisation prepared rapid gradient-echo sequence, field of view = 256 mm × 240 mm × 192 mm, matrix dimensions = 256 × 240 × 160, 1-mm isotropic resolution, repetition time = 2250 ms, inversion time = 900 ms, echo time = 2.99 ms, and flip angle = 9°.

### 2.5 Sensor–space analysis

#### 2.5.1 Pre-processing

MEG data were subjected to spatio-temporal signal–space separation (SSS) implemented in the Maxfilter software (Version 2.2.12) of Elekta Neuromag to remove noise generated from sources distant to the sensor array (Taulu & Kajola, 2005;Taulu & Simola, 2006). The Maxfilter procedure included movement compensation (locations recorded every 200 ms), bad MEG channel interpolation, and temporal SSS extension (with default buffer length 10 s and sub-space correlation limit 0.98). The origin in the head frame was chosen as (0,0,45) mm.

The following steps of analysis were performed in MNE-Python software package (Version 1.4.0,https://mne.tools/1.4/index.html) (Gramfort et al., 2013). After visual inspection of the raw data, bad EEG channels were interpolated. A notch filter at 50 and 100 Hz was then applied, followed by a low-pass filter with cut-off frequency 140 Hz. In order to remove eye movement artefacts, an Independent Component Analysis (ICA) was computed, removing a maximum of two ICA components. Our ICA procedure closely followed the examples provided for the MNE-Python software (https://mne.tools/stable/auto_tutorials/preprocessing/40_artifact_correction_ica.html), which uses the temporal correlation between ICA components and EOG channels as a criterion for the removal of ICA components.

#### 2.5.2 Frequency- and time-domain analyses

First, data of the four 60-s runs (with fade-in and fade-out periods removed) were averaged in the time domain to improve SNR, and a Fast Fourier Transform (FFT) with frequency resolution$0.01\bar{6}$Hz was applied. We divided the FFT spectrum into 10 segments of +/- 0.35 Hz centred at the frequency of interest and its 9 higher harmonics, which included 21 frequency samples at each side of the centre frequency. These segments were summed up for 10 harmonics. While some previous studies determined the number of summed harmonics based on their individual Z-scores (e.g.,Lochy et al., 2015), others also used a fixed value (Pescuma et al., 2022). Since we recorded from multiple sensor types (EEG, magnetometers, gradiometers), and in particular since we did not have a priori predictions about peak MEG sensors for word categorisation responses to determine Z-scores for the selection of harmonics, here we also chose a fixed value. Note that the selection of possibly non-significant harmonics may still lead to an increase of SNR due to the cancellation of summed noise, and adding higher harmonics has an increasingly smaller effect due to the approximate 1/f characteristic of the EEG/MEG frequency spectrum (i.e., lower amplitudes at higher frequencies).

For the word-selective frequency at 2 Hz, multiples of the base frequency (i.e., 10 Hz and 20 Hz) were excluded. In order to correct for the variations in baseline noise levels around each frequency of interest, the amplitude of 10 neighbouring frequency bins on each side of the centre frequency was averaged and subtracted from each frequency bin (Retter et al., 2020). A gap of one frequency bin on each side of the target frequency was included to avoid spectral leakage. The minimum and maximum values were also removed from the baseline interval. We applied the same summing and baseline correction procedure to the harmonics of the base frequency. Finally, Z-scores for word-selective and base frequencies were computed by dividing the baseline-corrected amplitudes by the standard deviation of the neighbouring bins from the baseline correction procedure.

Sensor–space results are presented separately for the three sensor types employed in this study: EEG, gradiometers, and magnetometers. The EEG measures the electric potential in Volts, magnetometers the magnetic flux in Tesla, and gradiometers the magnetic flux gradient in two orthogonal planar directions in Tesla per centimetre. As there are two gradient measurements per sensor location, the values of each gradiometer pair were plotted as the root-mean-square (RMS) per pair. Note that we present all results as Z-scores, which are comparable across sensor types.

We tested the reliability of our word-selective responses at the group level using cluster-based permutation testing, which is a common procedure to address the multiple-comparisons problem in EEG/MEG data (Maris & Oostenveld, 2007). The summed Z-score distributions across sensors at the centre frequency bin were subjected to two-tailed t-tests to first define clusters at a single-sensor p-value threshold of 0.05, and then the significance of clusters was established in a permutation procedure (10,000 permutations) and with a cluster p-threshold of 0.05.

The laterality of sensor–space responses was investigated by focusing on EEG for comparison with previous FPVS studies, as well as on gradiometers which allow a closer association between peaks in the distribution and the putative location of sources underneath those sensors than EEG and magnetometers (Hämäläinen et al., 1993). For EEG, we selected electrodes PO7 and PO8 in left and right posterior scalp regions, respectively, as they were the only electrodes that overlapped between our EEG setup in the MEG and the previous study byLochy et al. (2015). Note the neighbouring EEG electrodes are sensitive to similar brain structures due to volume conduction. Since no comparable data from MEG studies were available, we chose pre-defined groups of left and right temporal gradiometers and used their respective averages (options “Left-temporal” and “Right-temporal” from MNE-Python’s function “read_vectorview_selection”).

### 2.6 Source space analysis

Source estimation was performed on combined EEG/MEG data using L2-minimum-norm estimation, as appropriate for data where the number and location of sources are not known a priori (Hämäläinen & Ilmoniemi, 1984;Hauk et al., 2022). In the frequency domain, source estimates were computed for the summed topographies across harmonics for the word-selective and base frequencies, respectively. This was performed in MNE-Python software with standard parameters settings. We used individual MR images for head modelling. The MRI data were pre-processed in Freesurfer V6.0.0 (Fischl, 2012), and the head model (three-layer boundary element model) created in MNE-Python. We used L2-minimum-norm estimation without depth weighting or noise normalisation, and with a loose orientation constraint (ratio of variances between tangential and normal dipole components: 0.2). We used a regularisation parameter based on an SNR value of 3 (default in the software). The procedures for baseline correction and Z-scoring in the frequency domains as described above were also applied to source space results.

The significance of word-selective responses in source space was determined analogously to our procedure in sensor space using cluster-based permutation testing. Laterality in source space was examined by collapsing Z-scores across most of the temporal lobes, by combining the regions-of-interest (ROIs) inferior, middle, and superior temporal lobe from the Desikan–Killiany cortical parcellation in the left and right hemispheres, respectively (https://surfer.nmr.mgh.harvard.edu/fswiki/CorticalParcellation).

## 3 Results

**Fig. 2.**
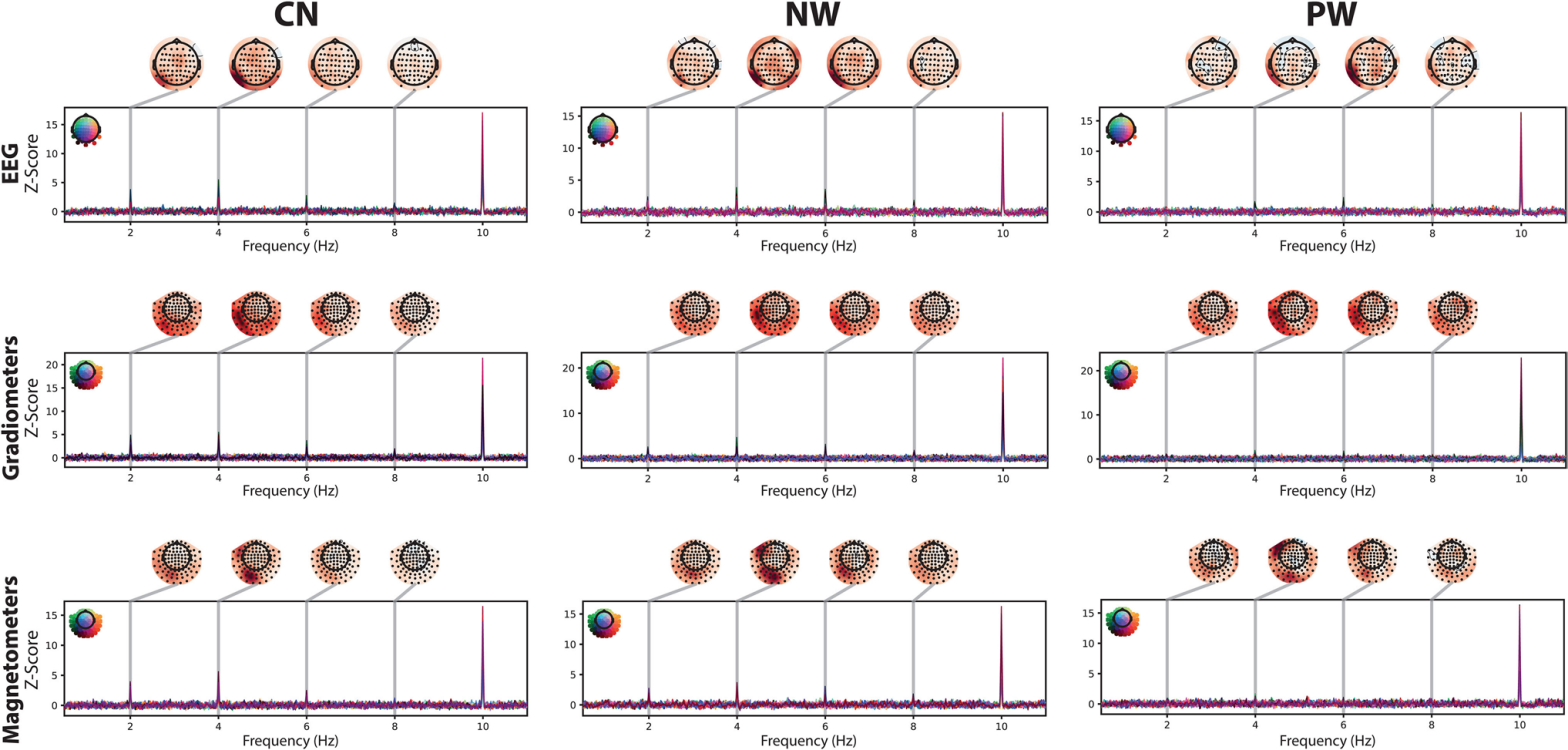
Z-scored spectra for all conditions. Each panel shows the Z-scored frequency spectra for individual channel types (EEG, gradiometers, magnetometers) used in our EEG/MEG recordings between 0.5 and 11 Hz. The peak at 10 Hz corresponds to the base frequency of visual stimulus presentation, and the peaks at multiples of 2 Hz correspond to responses at the word-selective frequency and its harmonics. Line colour indicates the position of the corresponding sensor in the sensor array (inlet in top left of panels). The topographies at the top of each panel represent Z-score distributions for the first four harmonics of the word-selective responses. CN: consonant strings; NW: non-words; PW: pseudo-words.

Figure 2shows the grand-averaged Z-scored frequency spectra for the condition in which words were interspersed among consonant strings (“CN”). The peak at 10 Hz reflects the base frequency, that is, the common response to all stimuli, while the peaks at the harmonics of 2 Hz represent word-selective responses, which discriminate between words and consonant strings in this example. The topographies of Z-scores are shown for the first four harmonics of the word-selective response and for each sensor type at the top of each panel. All sensor types show reliable word-selective responses in posterior sensors.

**Fig. 3.**
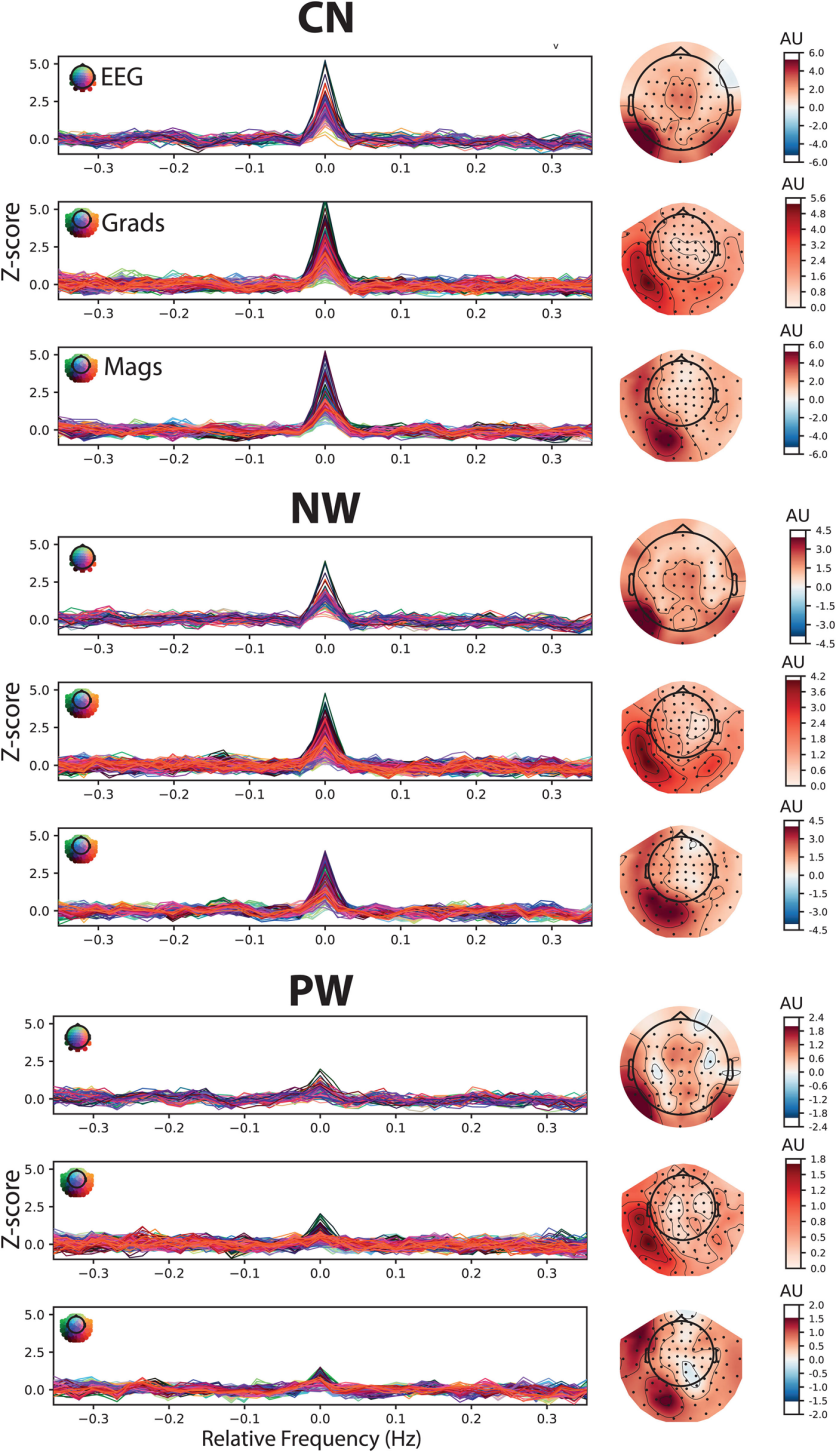
Z-scored summed frequency spectra around harmonics of word-selective frequencies. Each group of three panels presents results for one condition for all three sensor types. Each panel contains an overlay of all sensors (coloured lines) within EEG (top of each condition), gradiometers (“Grads”, middle), and magnetometers (“Mags”, bottom). The peak at 0 Hz for word-selective responses indicates reliable discrimination between words and base stimuli. Topographies on the right represent averaged Z-score distributions at the centre frequency. CN: consonant strings; NW: non-words; PW: pseudo-words.

Figure 3presents the Z-scored summed frequency spectra around harmonics of the word-selective frequencies for each condition (words among consonant strings, CN; words among non-words, NW; words among pseudo-words, PW). For each condition, all three sensor types are shown (EEG, gradiometers, magnetometers). While Z-scores are highest in the CN and lowest in the PW condition, a peak at the centre frequency (0 Hz in the summed epochs in the frequency domain) above the baseline indicates a reliable word-selective response at the group level in all conditions and sensor types. This is confirmed by the topographies on the right, which show the Z-score distributions for the summed spectra at the centre frequency. All topographies are characterised by left-lateralised peaks in posterior areas.

**Fig. 4.**
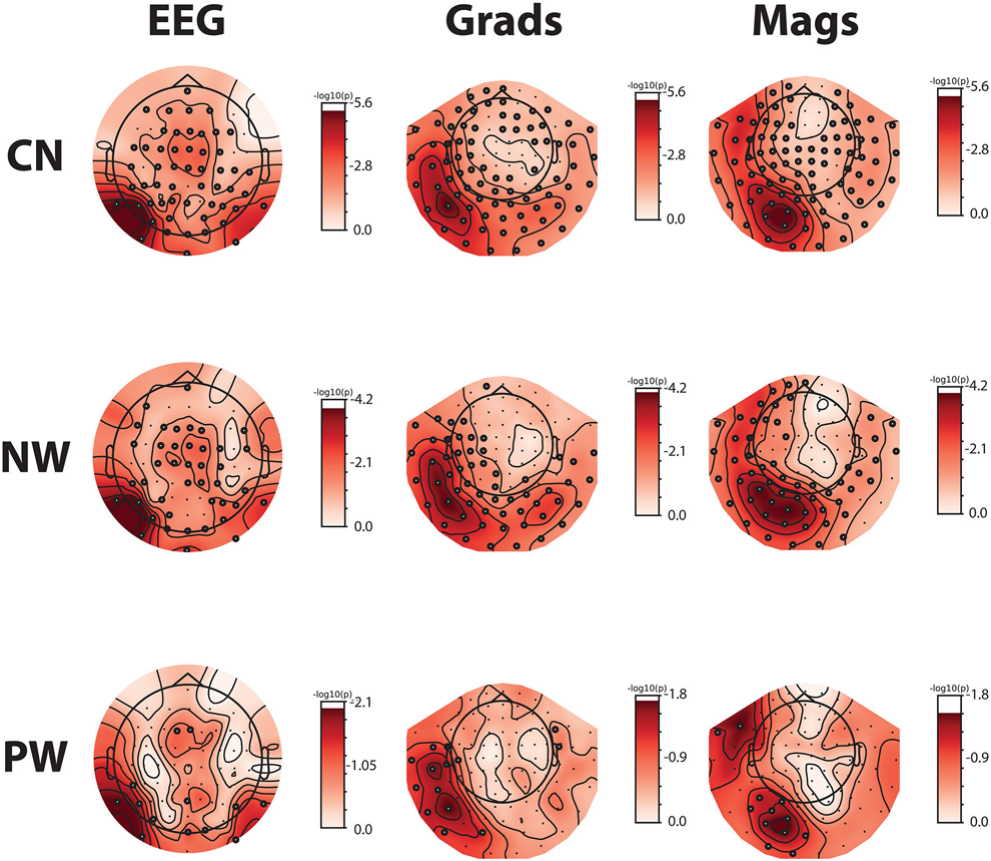
Statistics in sensor space. Summed Z-scored spectra were tested against zero using cluster-based permutation tests for each sensor type separately. Black circles indicate sensors that are part of a significant cluster. CN: consonant strings; NW: non-words; PW: pseudo-words.

Figure 4displays the corresponding cluster-based permutation results in sensor space for each condition and sensor type. Small black circles indicate sensors that are part of significant clusters. While the number of significant sensors decreases from CN over NW to PW, the dominant left-hemispheric peaks are significant in all cases.

This pattern of results is confirmed by our source space analysis inFigure 5. Here, Z-score distributions across the cortical surface obtained from L2-minimum-norm estimates were masked with the significant clusters from a cluster-based permutation test. Again, the extent of significant clusters follows the pattern CN>NW>PW, but several peaks in the left temporal lobe are significant in all conditions.

**Fig. 5.**
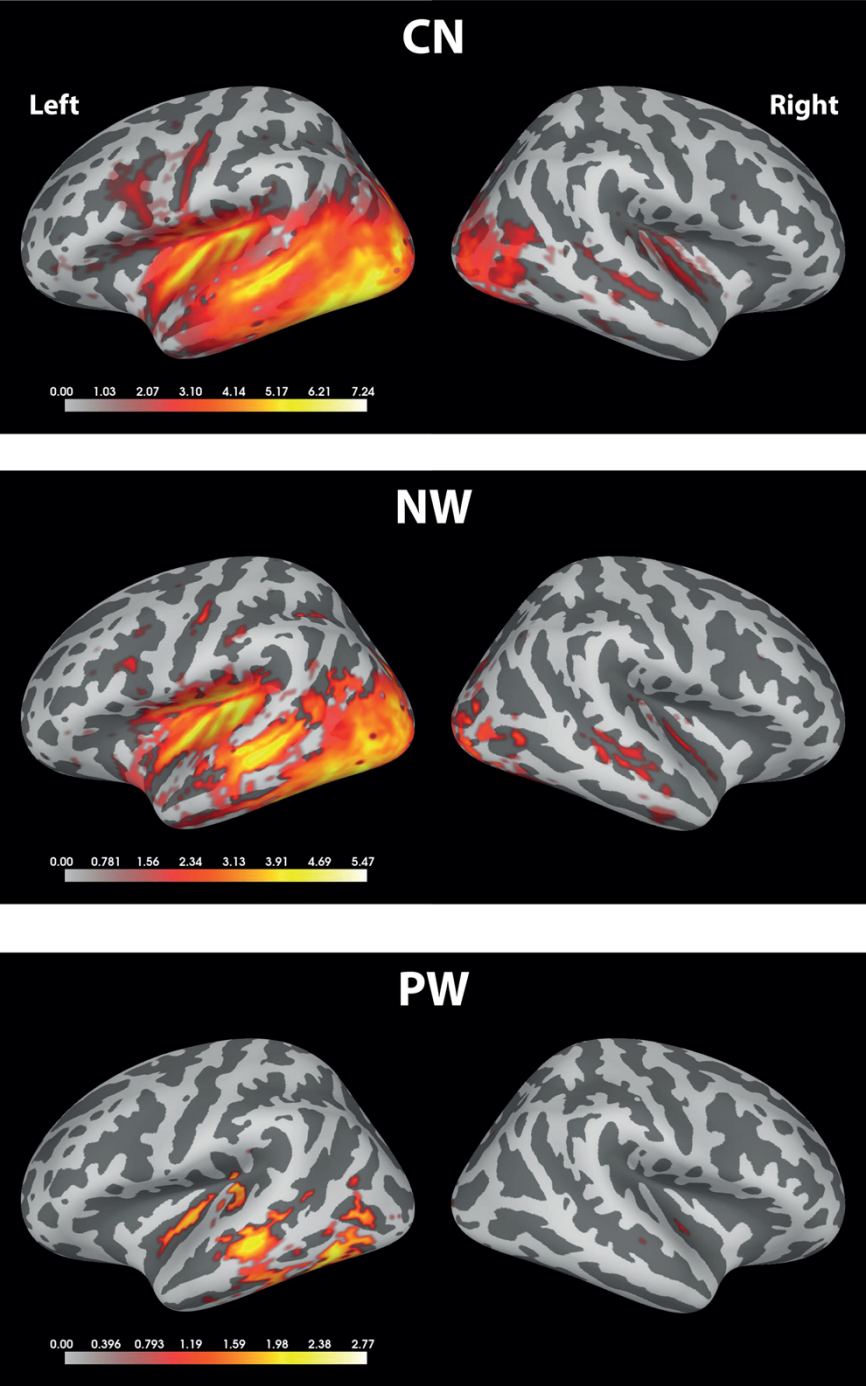
Source space analysis of word-specific responses. The panels show word-specific Z-scored summed spectra in source space (based on L2 minimum-norm estimation) masked by significant clusters from a cluster-based permutation test against zero. CN: consonant strings; NW: non-words; PW: pseudo-words.

In order to evaluate the robustness of word-selective responses and their laterality across individual participants, we show individual Z-scores for the selected occipito-temporal EEG electrodes and gradiometers inFigure 6. For each participant, a larger blue than orange bar indicates a left-lateralised word-selective FPVS response. While the Z-scores decreased from CN to PW as before, and they did not exceed Z > 2 for every participant, the majority of responses were left-lateralised, especially for gradiometers.

**Fig. 6.**
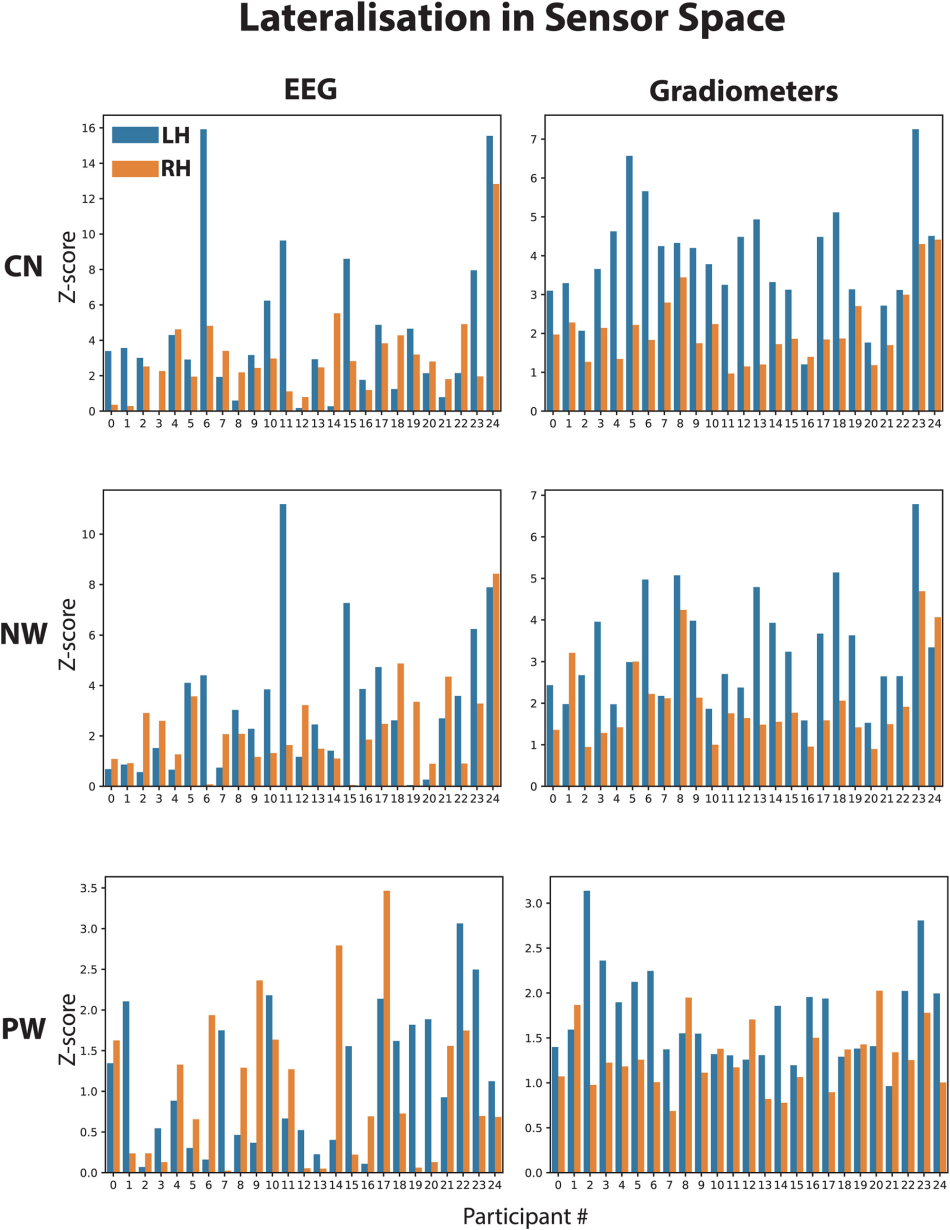
Z-scores for left and right sensor-space ROIs for individual participants. The bar graphs show average RMS Z-scores across left and right occipito-temporal EEG sensors (left) and temporal gradiometers (right), respectively. CN: consonant strings; NW: non-words; PW: pseudo-words.

We further quantified the laterality of these responses.Table 1presents the statistical results for a comparison of Z-scores between the two hemispheres, for EEG, gradiometers above temporal scalp regions, and for source estimates in the temporal lobes. For word-specific responses, gradiometers and source estimates produce reliably left-lateralised Z-scores for all three conditions. For EEG, word discrimination responses are numerically left-lateralised but non-significant, with decreasing Z-scores from CN to PW conditions. For the base response, only source estimates in the CN condition show a marginally significant lateralisation, and in this case to the right.

**Table 1.** Statistical results for laterality of Z-scores.

	EEG (PO7/PO8)Word-selective | base t(p)	Gradiometers (ROI)Word-selective | base t(p)	Temporal lobeWord-selective | base t(p)
CN	1.7 ( *0.11* ) | -1.6 (0.13)	7.0 ( 3.4e-7 ) | -1.6 (0.13)	6.4 ( 0.000001 ) | -1.9 ( 0.06 )
NW	1.4 ( *0.17* ) | -1.0 (0.33)	5.6 ( 0.00001 ) | -1.8 ( *0.08* )	4.2 (0.0003 ) | 0.0 (0.99)
PW	0.5 (0.62) | -0.4 (0.71)	3.4 ( 0.002 ) | -1.5 (0.16)	5.6 ( 0.000009 ) | -1.0 (0.32)

T- and p-values are shown for word-selective and base responses, respectively. (Marginally) significant p-values are underlined.

Table 2reports the percentage of participants who showed a numerically larger response in the left compared with the right hemisphere. The table also shows percentages for responses at the non-specific base frequency, as well as for source space results (seeFig. 7). These results mirror those ofTable 1, in that EEG only shows moderate left-lateralisation for word-selective responses in the CN condition, and close to bilateral responses in the NW and PW conditions. Nevertheless, for gradiometers, the degree of left-lateralisation is 68% or higher, reaching 96% in the CN condition. For the base responses, percentages indicate close to bilateral or right-lateralised responses, suggesting that lateralisation for word-specific responses is not the result of unspecific features of our paradigm.

**Table 2.** Percentage of participants with left-lateralised FPVS responses in sensor and source space for word-selective and base responses, respectively.

	EEG (PO7/PO8)Word-selective | base	Gradiometers (ROIs)Word-selective | base	Temporal lobeWord-selective | base
CN	60% | 44%	96% | 36%	88% | 44%
NW	52% | 52%	88% | 32%	76% | 52%
PW	52% | 56%	68% | 48%	96% | 48%

We present brain responses for ROIs in source space in the two separate hemispheres inFigure 7, with word-specific responses on the left and base frequency responses on the right. As apparent from the rightmost column inTable 2, at least 76% of participants show left-lateralised word-selective responses in all conditions. The PW condition is the most left-lateralised condition at 96%. In contrast, the base responses show left-lateralisation at or below 52%.

**Fig. 7.**
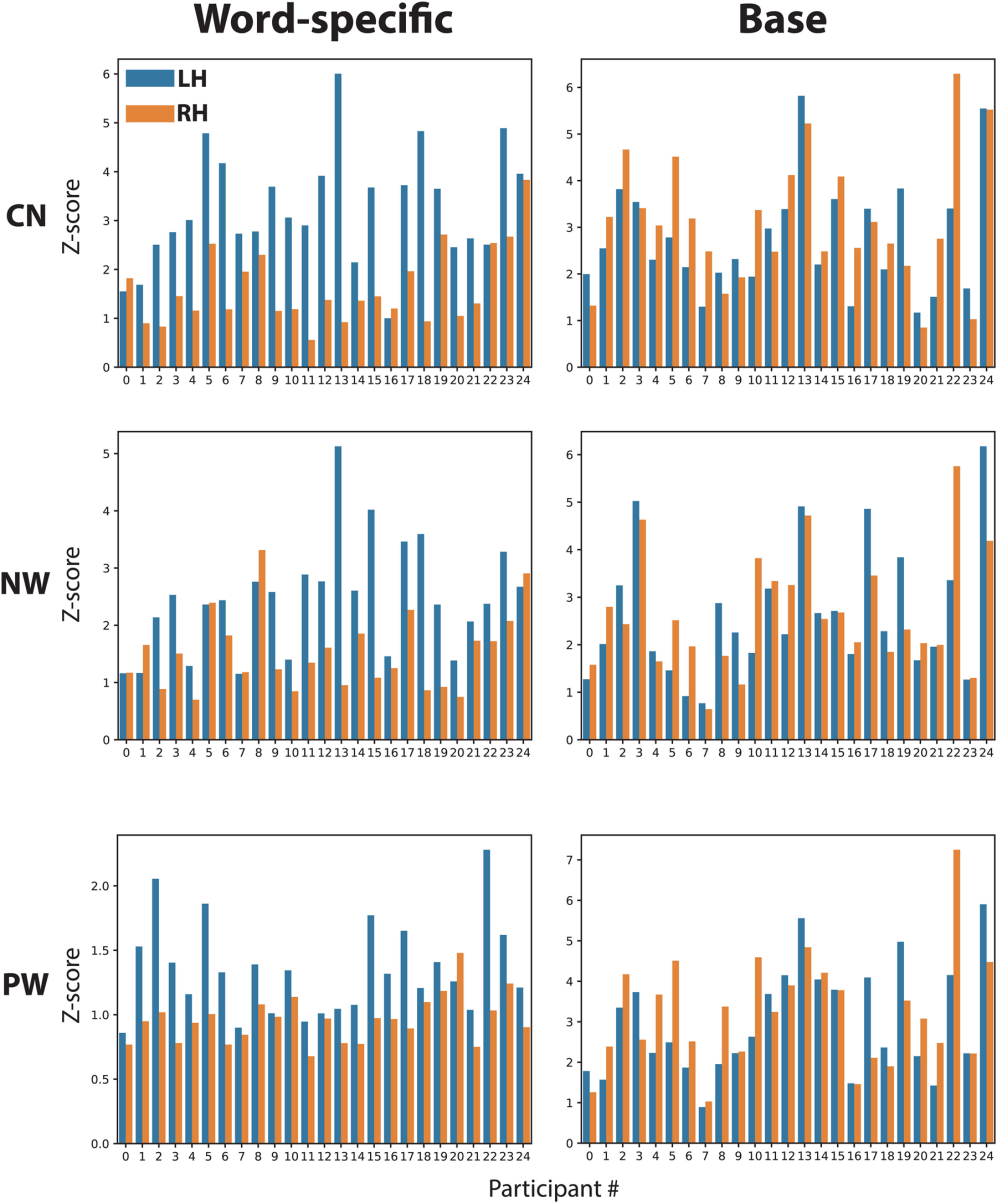
Z-scores for left and right temporal lobe ROIs in source space for individual participants. The bar graphs show mean absolute Z-scores averaged across inferior, middle, and superior temporal ROIs from the Desikan–Killiany cortical parcellation for individual participants (labelling as inFig. 6). Blue bars represent values for the left hemisphere (LH) and orange bars for the right hemisphere (RH), respectively. The left side shows bar graphs for word-specific responses and the right side for responses at the base frequency. CN: consonant strings; NW: non-words; PW: pseudo-words.

## 4 Discussion

We used fast periodic visual stimulation (FPVS) with combined EEG and MEG to study rapid word discrimination processes in the human brain. Our results showed reliable and mostly left-lateralised word-selective responses in all conditions at the group level, even for the most fine-grained lexical contrast where words were embedded in orthographically matched pseudo-words. Word-selective responses were modulated by the word-likeness of base stimuli, as they were strongest when words were presented among consonant strings, weaker among non-words, and weakest among pseudo-words. Therefore, we extend for the first time the results originally obtained in French (Lochy et al., 2015) to the English language.

In the past decade, EEG coupled with FPVS has shown substantial advantages in terms of sensitivity, recording neural responses with a high SNR in just a few minutes, and objectivity, by identifying and quantifying the response at a pre-determined frequency, without any explicit linguistic task. The oddball FPVS design (reviewed inRossion et al., 2020) measures neural responses to a deviant category of stimuli as an index of a*differential*processing between base and oddball stimuli (i.e., all common processes between base and oddball stimuli project to the common base rate response, which can be also objectively identified in the EEG spectrum). This method has been widely used with children learning to read (de Ghelcke et al., 2020,^2021^;Lochy et al., 2016;Lutz et al., 2024;Wang et al., 2023,^2024^), in developmental disorders (e.g., dyslexia (Lochy, Collette, Rossion, Schiltz, submitted)), and in healthy populations to study automatic lexical recognition (Lochy et al., 2015,^2024^;Marchive et al., accepted) or semantic discrimination (Volfart et al., 2021).

This approach provides important information about the neural bases of reading and left hemispheric lateralisation. Previous FPVS-EEG studies have shown left occipito-temporal topographies for word recognition, modulated by the word discrimination contrast (Lochy et al., 2015,^2018^,^2024^) and reading abilities (Lochy et al., 2016;Marchive et al., accepted;Wang et al., 2023). These findings are in agreement with the known brain regions implicated in reading and visual word recognition, such as the left ventral occipito-temporal cortex (lateral fusiform gyrus/occipito-temporal sulcus) (Cattinelli et al., 2013;Dehaene et al., 2005;Schurz et al., 2015;Wandell & Le, 2017), and align with fMRI studies showing left dominance in the VOTC for pre-lexical or lexical processes (Jobard et al., 2003;Vinckier et al., 2007), notably in the visual word form area (VWFA) (Cohen et al., 2000,^2002^;Dehaene et al., 2005,^2010^). In the context of our FPVS results, it is important to note that fMRI studies of word recognition invariably show some degree of bilateral yet asymmetric vOT responses to written words and letter strings (Behrmann & Plaut, 2015), a pattern that is mirrored in case-series studies of patients with damage to these regions (Behrmann & Plaut, 2014;Roberts et al., 2013). Thus, an approach involving implicit, rapid, and objective measurement is important to extend to other languages. Although word-selective responses were found in German (Aristei et al., 2017), a recent study encountered difficulties replicating these effects in 10 English participants (Barnes et al., 2021).Lochy et al. (2024)discussed this discrepancy as potentially stemming from linguistic differences between French and English in orthographic neighbourhood density (N), or methodological differences in the choice of pseudo-words.

In our study, we employed a larger number of participants and selected pseudo-words matched pairwise to the word stimuli for consonant/vowel structure, bigram frequency, and neighbourhood density. We successfully extended results from the French studies (Lochy et al., 2015,^2024^) to English. This is important, as previous replications with English stimuli had limited success (Barnes et al., 2021), which might have been due to intrinsic language differences. English and French languages vary in several orthographic properties, such as transparency, or the number of orthographic neighbours. English words have a greater orthographic neighbourhood size than French words overall, and neighbourhood size has been shown inconsistently to play a facilitatory (Grainger, 1992;Perea, 2015) or inhibitory (Perea et al., 2008;Pollatsek et al., 1999;Whitney & Lavidor, 2005) role on word recognition, although the picture is further complicated by both frequency effects and task demands (Grainger & Segui, 1990).

In our study, we found a word-selective response for all conditions at the group level, modulated by the word-likeness of base stimuli (mirroring behavioural effects found previously in behavioural lexical decision studies (Evans et al., 2012)). As explained byLochy et al. (2015), the word discrimination amplitude depends on the nature of the list context. For example, when words are discriminated among pseudo-letters, the response is larger and more bilateral (although with a left hemispheric dominance) than for words among non-words (NW) or pseudo-words (PW) (Lochy et al., 2015). Indeed, discriminated against pseudo-letters, responses to written words may reflect not only lexical and orthographic processes but also, more basically, the contrast of real letters to non-existing letter-like forms, thus generating more bilateral, lower-level, visual responses. Similarly, words among NW appear to trigger a larger amplitude response than words among PW (Lochy et al., 2015), possibly because this coarser contrast may be based on pre-lexical orthographic plausibility detection by comparison with the finer contrast. Our EEG/MEG results also evidence this modulation of response strength, with stronger responses for words among consonant strings, than among non-words or pseudo-words. Importantly, word-selective responses at the group level were located over the left vOTC.

Besides extending the results fromLochy et al. (2015)to the English language, we also presented MEG in addition to previous EEG results, and used their combination for source estimation. Only one previous study used EEG and MEG with an FPVS paradigm, on face categorisation (Hauk et al., 2021), and provided useful information on distributed sources over left and right hemispheres. Our source space results complemented those in sensor space. Word-selective responses in the group analysis were plausibly localised to the temporal lobe and left-lateralised. As in sensor space, they were most reliable for words in consonant string context, followed by non-words and pseudo-words. Significant Z-scores spread along the left temporal lobe for words embedded in a sequence of consonant strings showing a coarser discrimination contrast with a more bilateral response over the occipital visual areas. More focal and left-lateralised responses appeared with finer-grained lexical discrimination. However, the differences in SNR between conditions make a more detailed comparison of source distributions difficult. Nevertheless, the peaks of these distributions in the left temporal lobe were in similar locations across conditions, suggesting that the underlying distributions are similar and mainly differ with respect to their noise levels (see alsoLochy et al., 2018).

Second, we were able to assess individual discrimination responses. Previous studies on face-selective FPVS responses suggested that reliable category-specific responses could be obtained for the majority of participants with only a few minutes of data recording, raising the possibility that FPVS could become a standard tool for localiser scans or to monitor perceptual and cognition processes in clinical populations (Lochy et al., 2015;Rossion et al., 2015). With respect to word-like stimuli, the study byLochy et al. (2015)reported that word-specific responses for French words among well-matched pseudo-words could be detected as above baseline for the majority of participants, especially when topographies and laterality were taken into account. This high rate of response detection at the individual level was recently replicated (Lochy et al., 2024). Such a high sensitivity is an extremely important factor for future investigation of clinical populations (e.g., dementia) and developmental disorders (e.g., dyslexia) or for assessing the emergence of brain specialisation for print and lexical representations in young children. Interestingly, in the present study, MEG and source estimation produced a more consistent pattern with respect to lateralisation of word-selective responses than EEG. While EEG produced only numerically left-lateralised but non-significant responses at the group level, gradiometers achieved more than 68% and up to 96%, respectively, and in source space reached its highest level at 96% in the pseudo-word condition.

While we found clear left-lateralisation for word-specific responses in gradiometer signals and in source space, our EEG signals were generally more bilateral. In principle, this could be explained by differential sensitivity of different sensor types to different aspects of the underlying sources. MEG is relatively insensitive to radial sources, while those contribute strongly to EEG signals. Similarly, MEG sensitivity falls off more steeply with distance to the sensors than for EEG (and particularly for gradiometers). Thus, EEG may be sensitive to more widely distributed sources than MEG, which could be reflected in more bilateral responses. However, this is in contrast to previous studies that reported consistent left-lateralised responses for EEG as well (Barnes et al., 2021;Lochy et al., 2015;Pescuma et al., 2022). One major difference between these studies and the present study is that they had a higher density of EEG channels and analyses were performed on ROIs, while here we used only one EEG channel per hemisphere due to less dense electrode coverage. Thus, future studies should reveal whether this apparent discrepancy is due to stimulus material (e.g., English vs. French), differential sensitivities of MEG and EEG, or other methodological issues.

In conclusion, our study marks significant progress towards using MEG, potentially alongside EEG, to identify word-specific brain responses and their neural sources in individual participants. This approach could greatly enhance research across diverse participant groups, including clinical populations and children. Advancements in non-cryogenic on-scalp MEG sensors show particular promise, as they may further improve the signal-to-noise ratio (Boto et al., 2018).

## Data Availability

The data are available from the MRC Cognition And Brain Sciences’ data repository on request. The code used to analyse the EEG/MEG data is openly available on github:https://github.com/olafhauk/FPVS_WORDS.
